# Analysis of Failure Cause in Steel Wire-Reinforced Thermoplastic Composite Pipes for Sour Gas Field Water Transportation

**DOI:** 10.3390/ma18214865

**Published:** 2025-10-24

**Authors:** Zhiming Yu, Shaomu Wen, Jie Wang, Jianwei Lin, Chuan Xie, Dezhi Zeng

**Affiliations:** 1 Research Institute of Natural Gas Technology, PetroChina Southwest Oil & Gas Field Company, Chengdu 610213, China; 2 PetroChina Southwest Oil & Gas Field Company, Chengdu 610051, China; wensm@petrochina.com.cn (S.W.); snwangjie@petrochina.com.cn (J.W.); 3 Northeast Sichuan Gas District, PetroChina Southwest Oil & Gas Field Company, Dazhou 635000, China; linjw@petrochina.com.cn (J.L.); xiechuan@petrochina.com.cn (C.X.); 4 State Key Laboratory of Oil & Gas Reservoir Geology and Exploitation, Southwest Petroleum University, Chengdu 610500, China; zengdezhiswpu@163.com

**Keywords:** gas field water, H_2_S, wire-reinforced thermoplastic composite pipe, performance indicators, failure analysis, protection

## Abstract

Steel-reinforced thermoplastic pipe is widely used for water transportation in sour gas fields. However, under the combined effects of corrosive media, internal high pressure, and long-term environmental aging, premature failures such as leakage and bursting often occur. To clarify the failure causes and primary contributing factors of the composite pipes, this study conducted a comprehensive analysis through microscopic morphology examination of different typical failure cases, differential scanning calorimetry, Fourier transform infrared spectroscopy, and mechanical property testing. The main failure mechanisms were investigated, and targeted protective measures are proposed. Key findings reveal that the typical failure modes are ductile cracking, aging-induced brittle cracking, and aging creep cracking. These failures follow a mechanism of degradation of the inner and outer polyethylene protective layers, penetration of the medium and corrosion of the steel wires, reduction in pressure-bearing capacity, and eventual structural damage or leakage propagation through the pipe wall. Notably, oxidation induction time values dropped as low as 1.4–17 min—far below the standard requirement of >20 min—indicating severe antioxidant depletion and material aging. The main controlling factors are poor material quality, external stress or mechanical damage, and long-term aging. The polyethylene used for the inner and outer protective layers is critical to the overall pipe performance; therefore, emphasis should be placed on evaluating its anti-aging properties and on protecting the pipe body during installation to ensure the long-term safety and stable operation of the pipeline system.

## 1. Introduction

In recent years, with the increasing intensity of oil and gas exploration, the volume of produced water from gas fields has been rising annually. Effective management of produced water transportation is crucial for ensuring the safe and environmentally friendly development of gas fields. Due to the common presence of highly corrosive media such as H_2_S, CO_2_, and Cl^−^ in gas field water, metal pipelines used for transportation are highly prone to severe corrosion [1,2,3]. Non-metallic materials, owing to their excellent chemical inertness, have gained wide attention and application in oil and gas fields. Non-metallic and composite pipes offer significant advantages, including corrosion resistance, light weight, and ease of transportation and installation [4], proving effective in mitigating pipeline corrosion and reducing operational costs. Statistics indicate that non-metallic pipelines currently account for approximately 10–20% of total surface gathering and transportation pipelines. In the early stages, glass fiber reinforced plastic (GRP) pipes were predominantly used; however, due to their inherent brittleness and susceptibility to fracture, steel wire-reinforced thermoplastic composite pipes (SRTPs) and flexible composite high-pressure transportation pipes—with their superior flexibility—have become more suitable for mountainous and undulating terrains. As manufacturing technologies have matured and costs have decreased, the application of SRTPs has grown rapidly in recent years [5,6]. Nevertheless, various new failure issues have increasingly emerged, leading not only to environmental pollution but also to disruptions in the safe operation of gas fields.

Failure types of non-metallic pipelines include pipe body failure, joint failure, and mechanical damage. Liao et al. [7] analyzed the failure of glass fiber-reinforced composite pipes for high-pressure sewage transportation and found that aging led to reduced resin hardness and elastic modulus, significant deterioration of the fiber–resin interfacial properties, and the formation of voids and cracks under internal pressure. Kong et al. [8] investigated the failure of reinforced thermoplastic pipes used in petroleum transportation systems, where the direct cause was the damage to sealing rings, resulting in leakage of the transported medium at the joints. Ding et al. [9] studied a bulging failure on the outer surface of a service-aged steel wire-reinforced thermoplastic pipe (SRTP) after six years, attributing it mainly to scratch defects on the inner surface and poor resin stability near the cracked areas. Zhang et al. [10] analyzed leakage failures in SRTP body sections near heat-fusion sleeve joints used for water transportation in oilfields, revealing that excessive welding of the sleeves damaged the pipe’s cross-sectional structure, allowing fluid to penetrate into the reinforcement layer, corroding the steel wires, reducing pressure-bearing capacity, and ultimately causing burst failure. Khalid et al. [11] reported that under high-pressure and high-temperature conditions with acidic gases (CO_2_, H_2_S) and hydrocarbons, the primary damage to polymer liners of non-metallic composite pipes was due to medium permeation. Owing to the strong heterogeneity, wide variety, large performance differences, and diverse service conditions of non-metallic composite pipes, their failure causes are often more complex [12,13,14]. In the field of water transportation in gas fields, steel wire-reinforced thermoplastic pipes are mainly used. During long-term service, SRTP may fail due to material property degradation, environmental and load coupling effects, among other factors [15,16,17], with cracking and leakage being the predominant forms. At present, with the increase in usage and service time, research reports on various failure modes are still limited, and the analysis of the main controlling factors of failure remains to be further improved.

SRTPs generally adopt a three-layer structure: the middle layer consists of a welded steel wire mesh skeleton, a steel plate mesh skeleton, or a helically wound steel wire skeleton, while the inner liner and outer protective layers are made of polyethylene (PE) resin. The steel wire is carbon steel wire with the following principal chemical composition: C 0.35–1.00%, Si 0.10–0.30%, Mn 0.30–1.20%, P ≤ 0.035%, S ≤ 0.035%, Cu ≤ 0.20%; its ultimate tensile strength is 1720–1970 MPa. Polyethylene provides excellent chemical resistance, flexibility, and processability; its performance requirements comply with SY/T 6662.1-2022 [18]. Necessary additives—such as antioxidants, UV stabilizers, and colorants—are incorporated to enhance aging resistance. Therefore, based on the material and structural characteristics, the main advantages of SRTPs are good flexibility and the ability to be manufactured in large diameters (up to 600 mm). Their drawbacks include susceptibility to damage from external mechanical forces, a risk of steel wire corrosion, and complicated repair.

Currently, there is no unified international standard specifically for SRTPs. For material selection and performance evaluation, the principal reference is SY/T 6662.1-2022 [18], which covers four aspects: polyethylene raw material requirements, steel reinforcement structure requirements, appearance and dimensional requirements, and pipe performance requirements. The main performance indicators are listed in Table 1. However, the purpose of this study was not to conduct a comprehensive qualification test of the pipes, but rather to perform a root cause analysis on pipelines that had already failed in service. To efficiently identify aging and failure modes of the pipes, this research focuses on employing methods such as microscopic morphology analysis, oxidation induction time (OIT), Fourier transform infrared spectroscopy (FTIR), and hardness testing. These techniques are effective in diagnosing key issues, including material degradation, oxidation extent, and interface failure. This paper systematically investigates failure cases of SRTP used in sulfur-containing gas field water conditions, reveals their failure modes and dominant influencing factors, and proposes targeted protective measures, with the aim of providing a theoretical basis and experimental support for improving the service safety and reliability of such pipelines.

## 2. Background and Methods

Taking the Sichuan & Chongqing gas fields as an example, the transported medium is primarily gas field water separated from gas–liquid mixtures, characterized by high salinity (average 50,000–100,000 mg/L), saturated H_2_S/CO_2_, pH of 4–7, and minor sediment content. Operating pressures range from 0.5 to 2 MPa, and operating temperatures from 20 to 40 °C. The polyethylene inner liner is in direct contact with the transported medium, and its performance directly determines the structural integrity of the entire composite pipe. Common specifications for steel-reinforced composite pipes in gas field water transportation are DN100–150 mm (Diameter Nominal), with pressure ratings of 2–6 MPa and temperature limits below 60 °C. The designed service life is ideally 30–40 years. The allowable operating temperature is ≤65 °C when the liner is made of polyethylene/high-density polyethylene, and ≤75 °C when made of heat-resistant polyethylene. The allowable operating pressure is subject to a reduction factor of 0.69–0.90 based on service temperature.

According to survey statistics, construction-related damages include scratches, dents, and abrasion on the outer wall caused by contact with hard rocks, as well as localized compression of the pipe due to large hard stones left at the bottom of the laying trench before backfilling. During service, failures primarily involve pipe body rupture and cracking, most frequently occurring at the 6 o’clock position on the pipe underside, with service durations ranging from several years to over a decade.

The microscopic morphology changes of the failed pipe were observed using a scanning electron microscope (ZEISS Sigma 300, Oberkochen, Germany). The molecular chain structure of the samples was analyzed with a Fourier transform infrared spectrometer (Bruker INVENIO R, Ettlingen, Germany) over a scanning range of 500–4000 cm^−1^. Oxidation induction experiments were conducted on each group of samples using a thermal analysis system (Mettler-Toledo TGA/DSC2, Greifensee, Switzerland) to determine the oxidation induction time (OIT). The inner wall hardness of the samples was measured with a Shore hardness tester (INNOVATEST FALCON507, Maastricht, The Netherlands). The composition of gas field water was analyzed using an ion chromatograph (ICS-6000, Thermo Fisher Scientific, Waltham, MA, USA). Experimental data were processed and plotted using OriginPro-2024 software.

## 3. Results Analysis

### 3.1. Burst Failure

**Figure 1:**
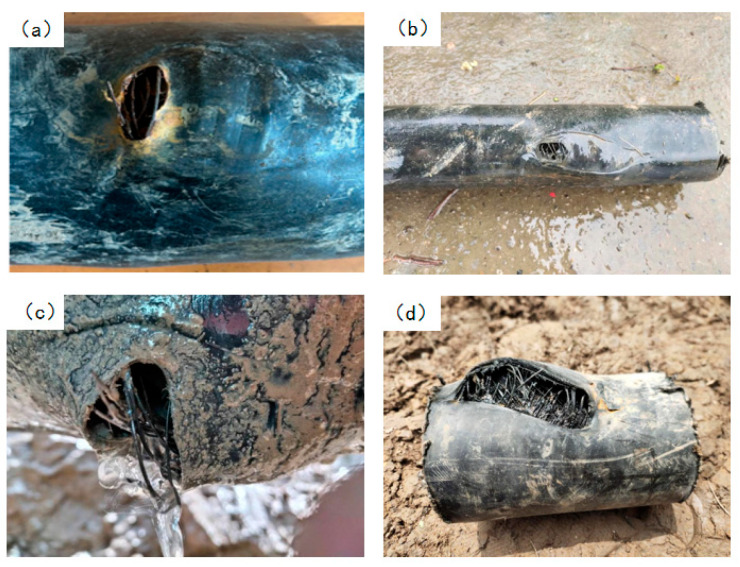
Morphologies of rupture and leakage on the underside of the pipe body: (**a**) First failure, (**b**) Second failure, (**c**) Third failure, (**d**) Fourth failure.

Case 1, in a gas well, the produced gas contained 4.6 g/m^3^ of hydrogen sulfide, with a daily water output of 280 m^3^. After gas–liquid separation, the gas field water was of the CaCl_2_ type, with a salinity of approximately 54,600 mg/L, a pH of 6.9, and a small amount of suspended solids. Steel-reinforced composite pipes were used to transport the water to an injection well. The pipe specification was DN100 (Diameter Nominal) × 11 (wall thickness) mm, with a design pressure of 6.3 MPa. The buried pipeline was 6.7 km in length; under normal operation, the inlet pressure was 2.9 MPa, the outlet pressure was 0.6 MPa, and the water temperature at the station outlet was 25–30 °C. Since commissioning in 2017, four instances of pipe body rupture and leakage have occurred, all located at the 6 o’clock position on the underside of the pipe in low-lying farmland sections. The failure mode in all cases was a circular bulging rupture, characteristic of local overpressure-induced burst failure, with exposed steel wires. The macroscopic failure morphologies are shown in Figure 1.

**Figure 2:**
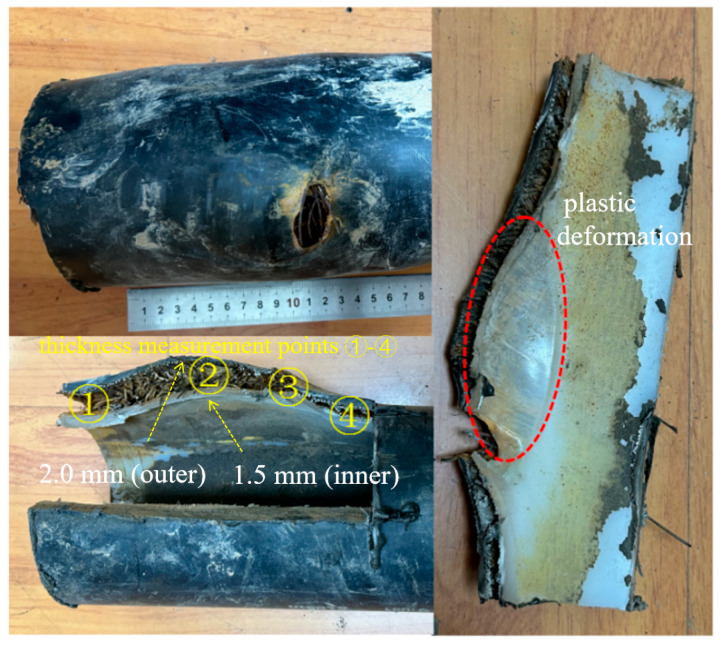
Morphology of rupture opening on the pipe body.

The first three failures exhibited similar morphologies. Taking the first case as an example for analysis, the rupture opening measured approximately 28 mm × 18 mm. Steel wires in the reinforcement layer were exposed in the failed area and showed clear signs of corrosion (Figure 2). At the fracture edges, both the outer and inner layers exhibited significant plastic deformation and wall-thickness reduction: the outer layer thickness decreased from 6.0 mm in the intact region to 2.0 mm in the failed region, while the inner layer thickness decreased from 7.0 mm to 1.5 mm, indicating that both layers ruptured due to overpressure. The intact regions of the pipe were relatively smooth and complete, while gray and brown loose deposits were observed inside the pipe.

**Figure 3:**
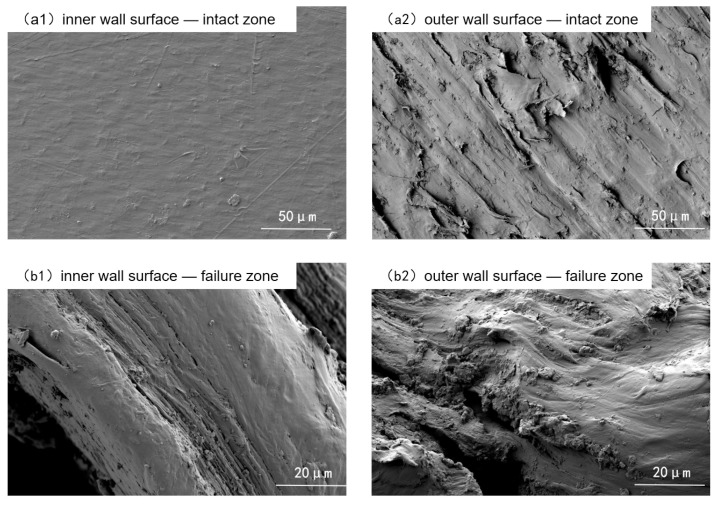
Micromorphology of inner and outer walls.

Microscopic examinations were conducted on the inner and outer walls of both failed and non-failed regions using scanning electron microscopy (SEM), as shown in Figure 3. The inner wall of the non-failed region was smooth and flat, with no obvious pores or cracks. In contrast, the outer wall of the non-failed region was rough, with distinct textures, scratches, and small pores and depressions, indicating a trend of aging and degradation. In the failed region, both the inner and outer walls exhibited groove-like cracks and prominent stretched-deformation textures.

**Figure 4:**
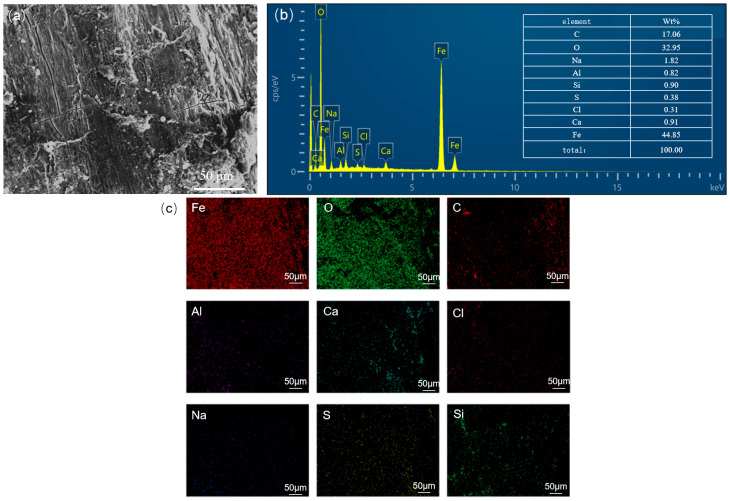
Corrosion morphology of steel wire corrosion products: (**a**) microscopic morphology, (**b**) elemental composition, (**c**) elemental distribution in the corrosion product layer.

Figure 4 shows the corrosion morphology of the steel wire skeleton and the elemental mapping of the corrosion products. Significant accumulation of corrosion products can be observed on the wires (Figure 4a). EDS results indicate that the main components are O (32.95 wt.%), Fe (44.85 wt.%), along with minor amounts of Ca, Si, S, etc. (Figure 4b). Elemental mapping of the corrosion layer also reveals that Fe and O are the predominant elements (Figure 4c), suggesting that the corrosion products are primarily iron oxides and that the steel wires have undergone relatively severe oxygen-induced corrosion. Given that the steel wire diameter is only about 1 mm, corrosion-induced thinning led to a sharp decrease in tensile strength, making fracture more likely and substantially reducing the local pressure-bearing capacity of the pipe.

**Figure 5:**
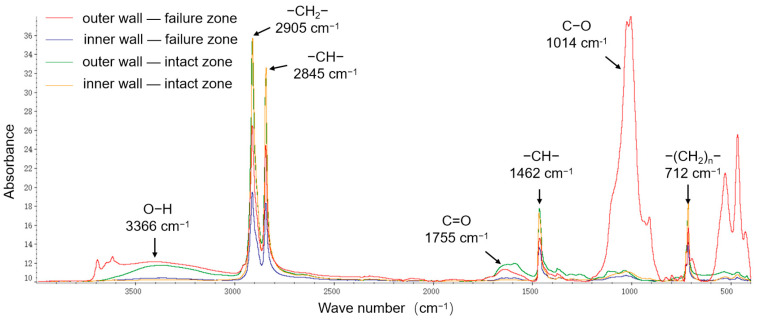
FTIR analysis.

FTIR was employed to analyze the chemical composition and variations in the primary functional groups on the inner and outer walls of the pipeline, as shown in Figure 5. The peaks at 2905 cm^−1^ and 2845 cm^−1^ correspond to the stretching vibrations of C–H bonds in the polyethylene molecular chain, while those at 1462 cm^−1^ and 712 cm^−1^ correspond to the in-plane and out-of-plane bending vibrations of C–H bonds, respectively. The C–H main chain signal intensity of the inner wall polyethylene was higher than that of the outer wall, indicating slight degradation on the outer wall. In the failed region’s outer wall, in addition to the main peaks from the polyethylene matrix, strong O–H (3366 cm^−1^) and C–O (1014 cm^−1^) signals were also detected. The O–H peaks originate from hydroxyl groups absorbed from water, the C=O and C–O peaks arise from oxidation of unsaturated double bonds in polyethylene. This indicates that significant oxidative degradation occurred in the outer wall of the failed region.

**Figure 6:**
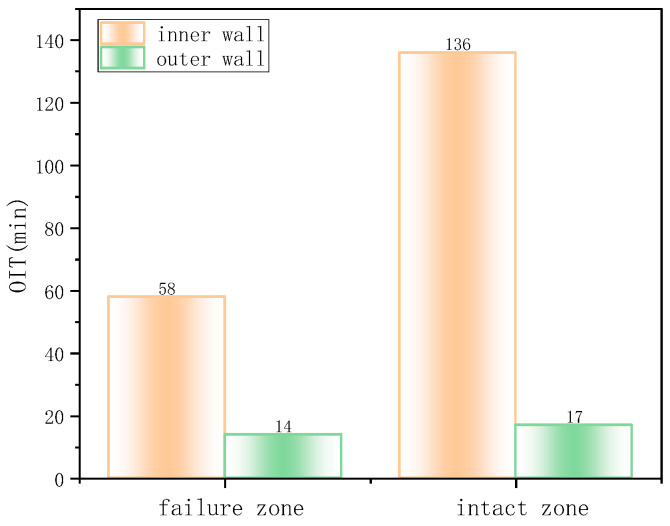
DSC oxidation induction time test results.

DSC was used to measure the OIT of the polyethylene material in the inner and outer layers, reflecting the degree of antioxidant depletion and hence the material’s antioxidative performance (Figure 6). The OIT of the inner wall polyethylene in the non-failed region was 186 min, and in the failed region, it was 141.8 min. In contrast, the OIT of the outer wall polyethylene was only 14 min for both failed and non-failed regions, far below that of the inner wall and failing to meet the SY/T 6662.1-2022 standard requirement of not less than 20 min (at 210 °C). Normally, polyethylene exhibits a slow aging rate, with an ideal service life of ~30 years. However, in this case, severe aging occurred in the outer wall after only six years of operation, indicating low initial antioxidative performance and poor material quality.

**Figure 7:**
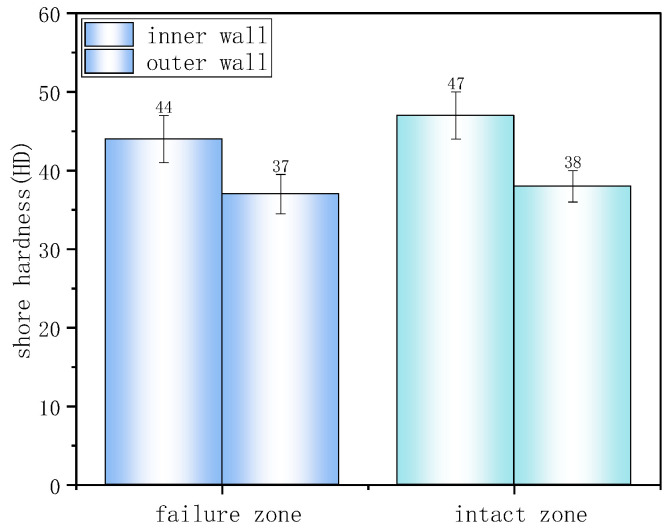
Shore hardness test results.

Shore hardness test results (Figure 7) showed that the hardness in both failed and non-failed regions of the inner wall was approximately 45 HD, higher than the outer wall’s 37 HD. Lower hardness reflects lower material crystallinity and weaker resistance to localized deformation damage such as indentation or scratching.

Based on the above analyses, the outer wall polyethylene material exhibited a rough surface with pore defects and signs of aging and degradation. FTIR and OIT results confirmed that the antioxidative performance of the outer wall polyethylene failed to meet the standard requirements and had undergone significant oxidative degradation. Severe oxygen corrosion occurred in the steel wire reinforcement. The primary failure mechanism was oxidative damage to the polyethylene matrix of the outer wall, allowing external corrosive media to penetrate into the pipe interior and cause corrosion damage to the steel reinforcement layer. This reduced the local strength and pressure-bearing capacity of the pipe. In addition, stress concentration at the pipe bottom under internal pressure and overlying soil load ultimately led to burst failure.

**Figure 8:**
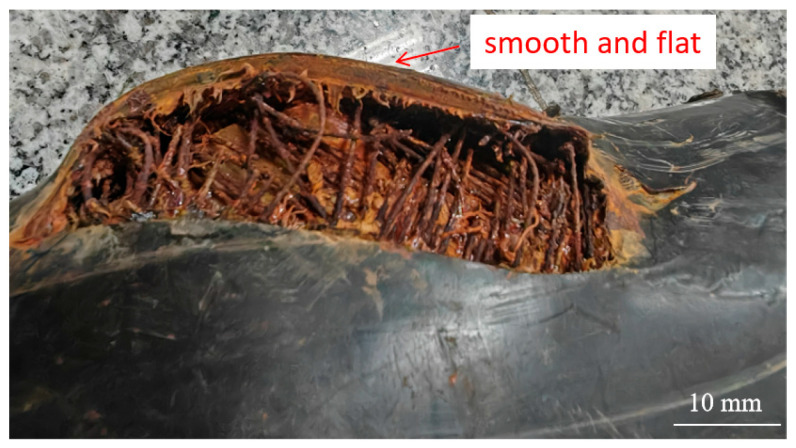
Cross-section formed by mechanical scratching.

For the fourth failure, the rupture opening was comparatively the largest, with a clean outer layer fracture (Figure 8) showing no significant plastic deformation or wall thinning. This indicates clear mechanical scratching, with the primary cause being severe mechanical damage during construction. Such damage accelerated aging and degradation of the outer layer during service, facilitated ingress of corrosive media into the interior steel wires, and ultimately led to burst failure.

Considering that all four failures occurred in low-lying farmland areas, it is inferred that the high soil moisture content in farmland facilitates the ingress of liquid-phase media through external damage sites into the pipe interior, triggering steel reinforcement corrosion and ultimately causing insufficient pressure resistance and failure.

### 3.2. Perforation and Leakage Failure

**Figure 9:**
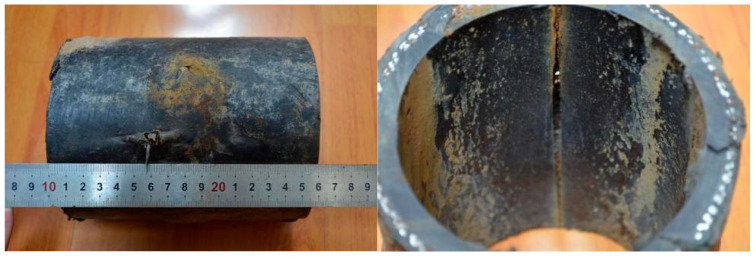
Macroscopic morphology of the outer and inner walls at the cracking failure site.

Case 2, a buried SRTP was installed downstream of a sulfurous gas well station. The pipe specification was DN100 mm, with a design pressure of 8 MPa, transporting gas-field water from the outlet of a gas–liquid separator. The hydrochemical analysis indicated that the gas field water was of the NaHCO_3_ type, with a salinity of approximately 24,306 mg/L and a pH of 7.2. The water contained dissolved H_2_S, giving it a characteristic rotten-egg odor. The pipeline operated at a daily temperature of about 50 °C, with a maximum pressure of ~6 MPa, and a daily water throughput of 300–500 m^3^. Commissioned in December 2010, the pipeline experienced cracking failure at the 12 o’clock position in November 2024, after nearly 14 years of service. Figure 9 shows the macroscopic morphology of the outer and inner walls at the cracking failure site. Two slight bulges with perforation leakage openings were observed on the surface. The polyethylene around the perforation edges exhibited thinning, curling, and deformation. Inside the pipe body, a longitudinal crack approximately 16 cm in length was found, with a maximum width of ~5 mm at the midpoint. The polyethylene along the crack showed no significant plastic deformation.

**Figure 10:**
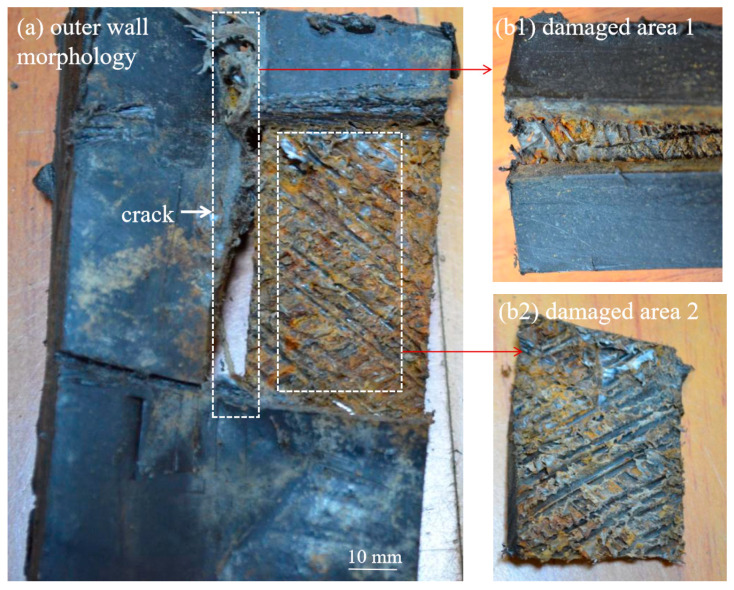
Macroscopic morphology after removal of the outer layer.

Figure 10 shows the macroscopic morphology after removal of the outer layer. In the central section of the longitudinal crack, the steel wire reinforcement layer was completely corroded, leaving only the bonding resin. Near the crack ends, steel wires were still visible, but all were completely fractured.

**Figure 11:**
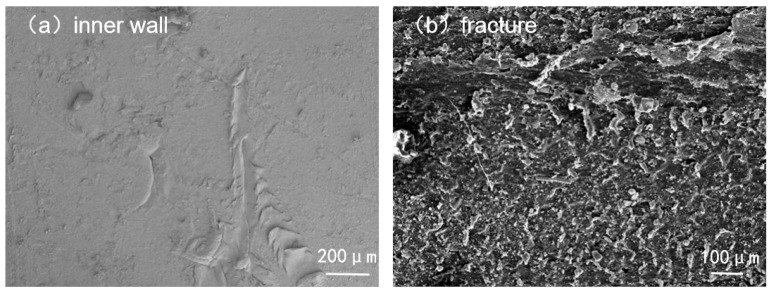
Micromorphology of the inner wall and fracture section.

Figure 11 presents the microscopic SEM morphology of the inner wall and fracture section. The inner wall exhibited groove-like scratches caused by erosion from solid sand and gravel particles. The fracture surface was relatively flat, with small flake-like or granular structures related to fracture in the crystalline regions of polyethylene, indicative of a brittle fracture mode.

**Figure 12:**
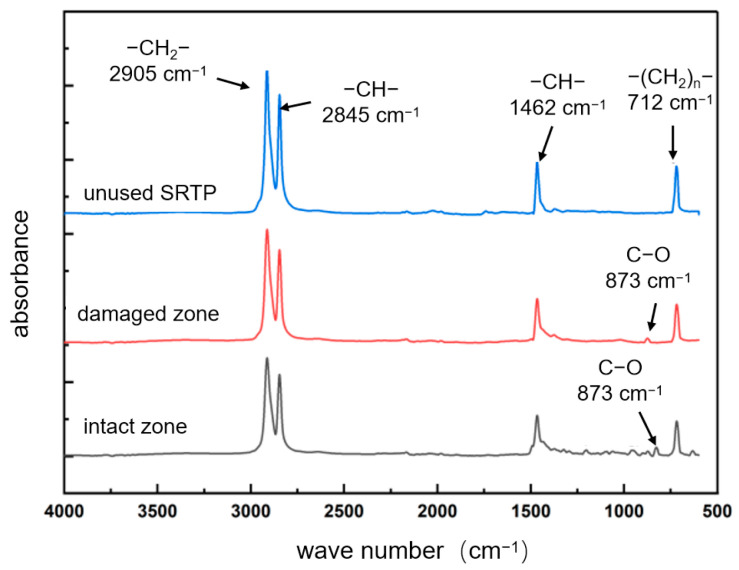
FTIR analysis results.

FTIR analysis results are shown in Figure 12. In the non-failed region, four characteristic absorption peaks were identified: at 2905 cm^−1^ (–CH_2_– saturated hydrocarbon stretching vibration), 2845 cm^−1^ and 1462 cm^−1^ (–CH– group vibrations), and 712 cm^−1^ (vibrations of –(CH_2_)ₙ– groups). In addition to these characteristic polyethylene peaks, the crack region also exhibited new peaks, such as a C–O stretching vibration near 873 cm^−1^. The presence of C–O bonds indicates oxidation of unsaturated double bonds in polyethylene, confirming that oxidative degradation occurred in the material near the cracking area.

**Figure 13:**
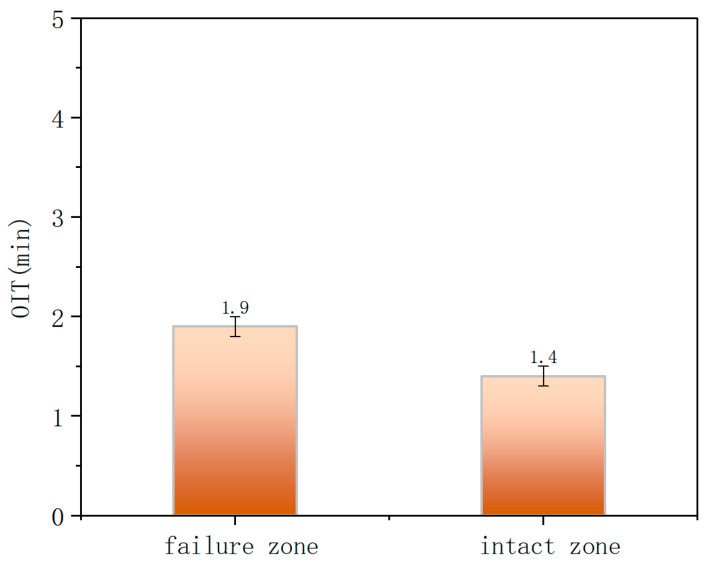
Oxidation induction time (OIT) measurement.

**Figure 14:**
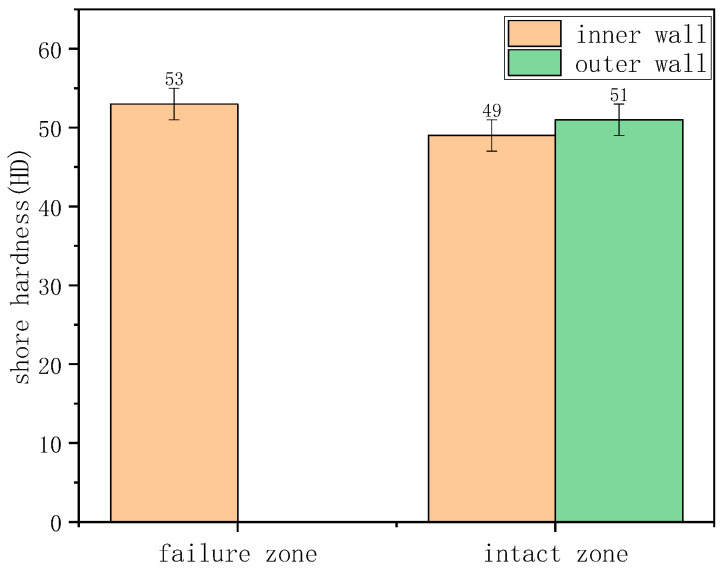
The average Shore D hardness values for the inner and outer walls.

DSC was employed to further characterize the depletion of antioxidants in the material (Figure 13). The OIT for the inner surface at the crack region and the non-crack region were 1.9 min and 1.4 min, respectively, both far below the SY/T 6662.1-2022 standard requirement of less than 20 min at 210 °C. This indicates that the material had undergone severe aging. Shore hardness test results are summarized in the table. The average Shore D hardness values for the inner and outer walls, in both the uncracked and cracked regions, were approximately 49–53 HD (Figure 14), with no significant differences observed.

In summary, morphological analysis revealed that the fracture surface exhibited a flat, brittle fracture feature. OIT testing demonstrated a severe reduction in the antioxidant capacity of the inner-wall polyethylene. The primary failure mechanism was aging-induced degradation of the pipe material during long-term service, leading to a loss of anti-aging performance. Under combined stress, the inner polyethylene layer first experienced environmental stress cracking, allowing gas-field water to penetrate into the intermediate steel wire reinforcement layer. This resulted in severe corrosion of the steel mesh, ultimately causing perforation and through-wall failure of the outer wall.

### 3.3. Bulging Deformation and Cracking Failure

**Figure 15:**
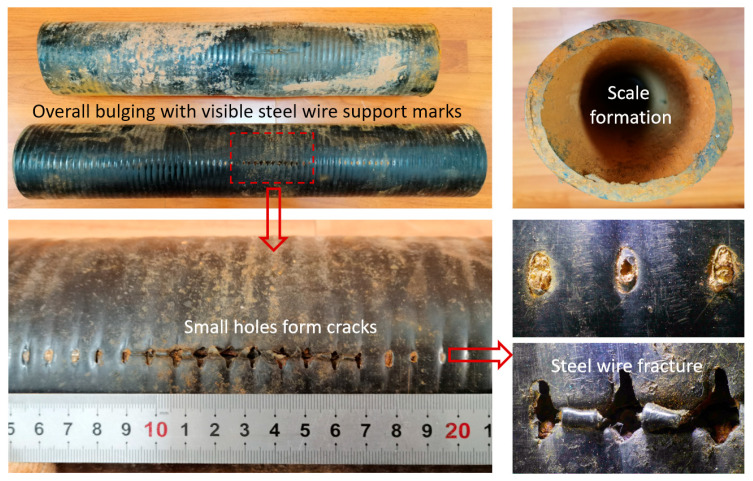
Macroscopic morphology of the failed pipe section and rupture area.

Case 3, an underground steel wire-reinforced polyethylene composite pipeline, approximately 2.7 km in length, with a specification of DN100 × 10 mm, a design pressure of 2.5 MPa, operating pressure of 2 MPa–2.2 MPa, water delivery rate of 43–45 m^3^/h, operated on average 3–4 h/day, with frequent start–stop cycles over the long term. This pipeline section was commissioned in 2006. Between 2020 and 2023, six incidents of rupture and leakage occurred within 200 m of the station outlet. Figure 15 shows the macroscopic morphology of the failed pipe section and rupture area. The entire pipe body exhibited bulging deformation, with the reinforcement steel wire mesh pattern visible. Multiple small perforations connected to form a longitudinal crack of approximately 15 cm in length. The reinforcement steel wires were exposed and showed severe corrosion, while large amounts of yellow scale deposits adhered inside the pipe.

**Figure 16:**
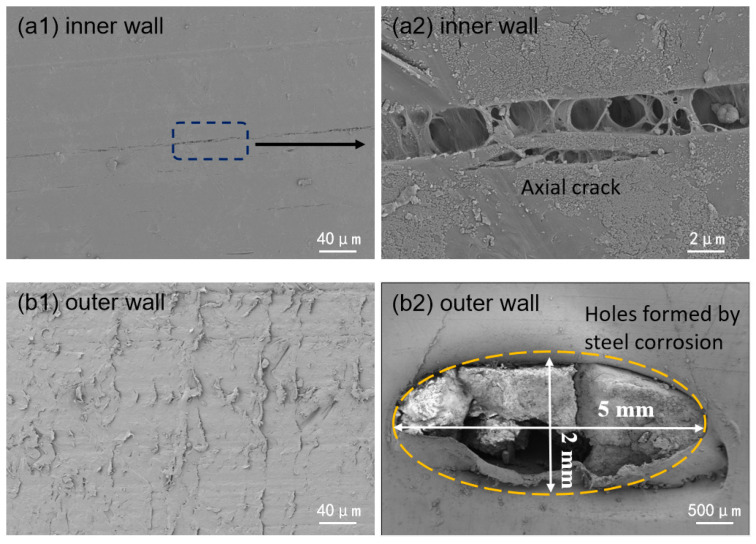
Micromorphology of the polyethylene inner and outer walls.

**Figure 17:**
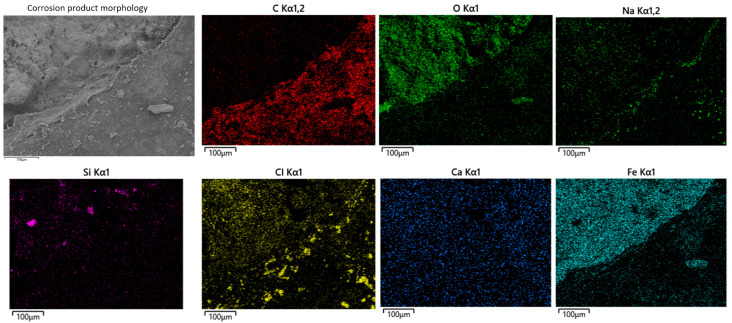
Corrosion morphology and elemental distribution of steel wire corrosion products.

**Figure 18:**
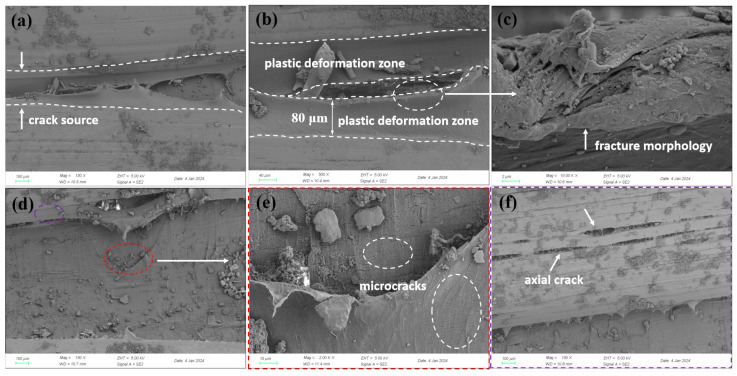
Micromorphology of the cracked inner wall region: (**a**) crack origin, (**b**) close-up of crack origin, (**c**) further magnified view showing micro-cracks, (**d**) crack branching, (**e**) magnified view of dense micro-cracks, (**f**) continuous axial micro-cracks.

Scanning electron microscopy (SEM) was used to analyze the microstructure of the inner wall. Figure 16 shows the microscopic morphology of the inner and outer polyethylene walls. In the undamaged area of the inner wall, multiple continuous axial cracks were observed in the polyethylene matrix, and signs of matrix degradation were present within the cracks. No visible defects were found in the undamaged areas of the outer wall, but the surface polyethylene matrix exhibited a certain degree of radial wear marks, an elliptical steel wire exposure area measuring approximately 5 mm in length and 2 mm in width was observed, and EDS analysis shows that the corrosion products of the steel wire are mainly iron oxides (Figure 17). In the cracked area, the inner wall was thinned, and a plastic deformation zone approximately 80 μm wide was observed along the crack edges, with signs of inward curling and tearing. Numerous axial microcracks were also detected within the crack zone, as shown in Figure 18. These may have originated from microcracks induced by matrix material aging, which subsequently propagated into transverse cracks under internal pressure. Transverse cracks then interconnected to form continuous cracks, ultimately leading to rupture under sustained internal pressure.

**Figure 19:**
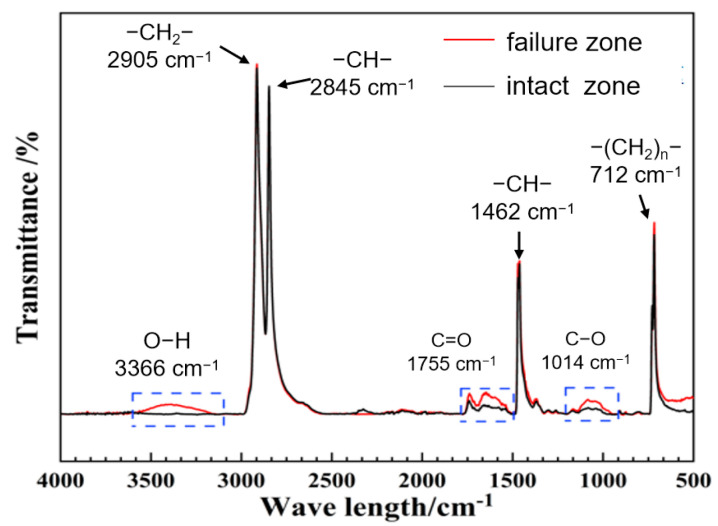
FTIR spectra analysis.

FTIR was employed to analyze functional groups in the inner-wall material of both failed and intact regions (Figure 19). The peaks at 2905 cm^−1^ and 2845 cm^−1^ correspond to the asymmetric and symmetric stretching vibrations of –CH_2_–, respectively; the peak at 1462 cm^−1^ corresponds to the bending vibration of –CH_2_–; and the peak at 712 cm^−1^ corresponds to the absorption band associated with multiple –CH_2_– linkages, representing the main chain of polyethylene molecules. In the failed region, stronger signals were detected for –OH (3366 cm^−1^) groups, as well as oxidation-related carbonyl C=O (1755 cm^−1^) and C–O (1041 cm^−1^) bonds, suggesting higher contents of these functional groups and indicating a more pronounced degree of aging.

**Figure 20:**
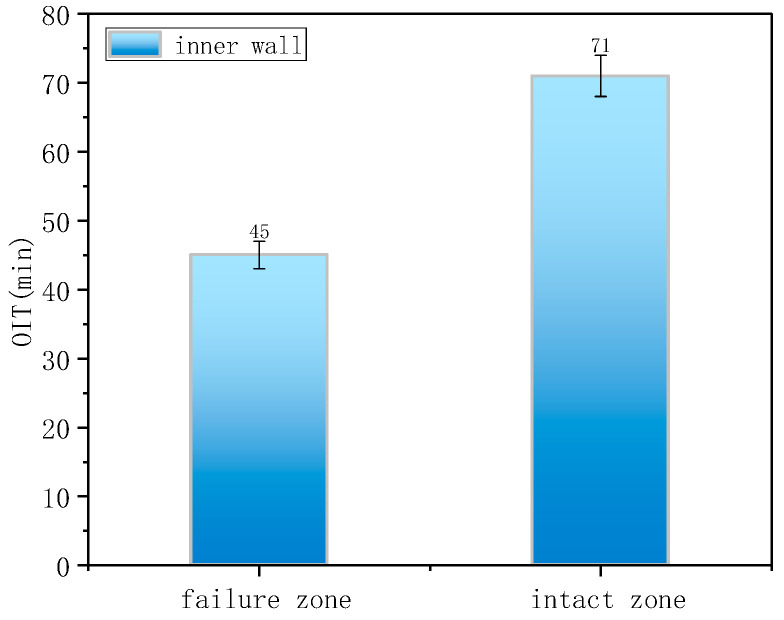
Oxidation induction time measurement.

**Figure 21:**
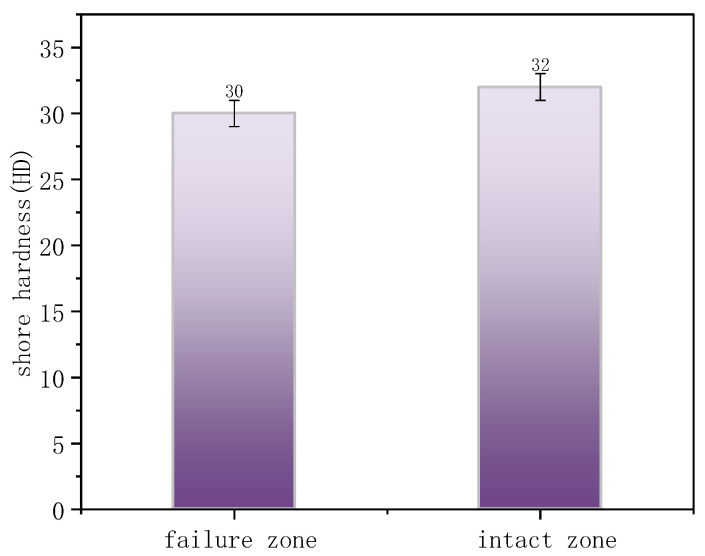
Shore hardness of the failure zone and intact zone.

The OIT values for the intact and failed regions were 71 min and 45 min (Figure 20), respectively, indicating that cracks preferentially initiated in areas with weaker oxidation resistance. Figure 21 presents the Shore hardness results for the inner wall. The hardness values for the failed and intact regions were 30 HD and 32 HD, respectively, whereas typical PE hardness values range from 45 to 55 HD. This significant reduction in hardness indicates material softening and reduced resistance to deformation.

In summary, the steel-reinforced composite pipe exhibited significant creep-induced bulging deformation, with continuous circumferential corrugations. The inner-wall PE developed numerous axial microcracks, accompanied by degradation of the molecular structure, reduced antioxidant performance, and decreased deformation resistance. Considering the operating conditions, 2.2 MPa operating pressure, close to the 2.5 MPa design pressure, and frequent start–stop cycles, the “water hammer effect” caused by valve operations could generate transient internal fluid pressure surges exceeding the design limit. The pipeline segment immediately upstream of the outlet was the most affected. The primary failure mechanism was aging and degradation of the PE matrix material. High-pressure operation and the water-hammer effect under frequent start–stop conditions accelerated creep deformation. Under coupled effects, continuous cracks formed on the inner surface, enabling environmental media to penetrate and corrode the steel wire reinforcement. Local stress concentration and internal pressure then promoted crack propagation, ultimately resulting in through-wall failure.

## 4. Discussion

Through the testing and analysis of the aforementioned three typical failure cases, the results reveal certain common patterns, providing critical evidence for identifying the root causes of failure. Firstly, in terms of material aging, FTIR detected distinct characteristic peaks of oxygen-containing functional groups in the failure zones of all cases, indicating oxidative degradation of the polyethylene molecular chains. DSC further confirmed that the oxidation induction time (OIT) values in Case 1 and Case 2 (as low as 14 min on the outer wall and 1.4 min in the inner wall crack zone) were far below the standard requirement (>20 min), demonstrating severe depletion of antioxidant capacity. Although the OIT value in Case 3 remained above the standard, the significant difference between the failed area (OIT 45 min) and the non-failed area (OIT 71 min) also clearly indicated a trend of aging degradation. Secondly, SEM observations revealed defects such as cracks, pores, and plastic deformation on both the inner and outer walls. Shore hardness testing also indicated material softening, confirming a significant decline in the barrier function of the polyethylene protective layer. Finally, EDS analysis of the steel wire skeleton showed that the corrosion products were primarily iron oxides. Severe corrosion and even fracture of the wires occurred due to media penetration, resulting in the complete loss of their reinforcing function.

**Figure 22:**
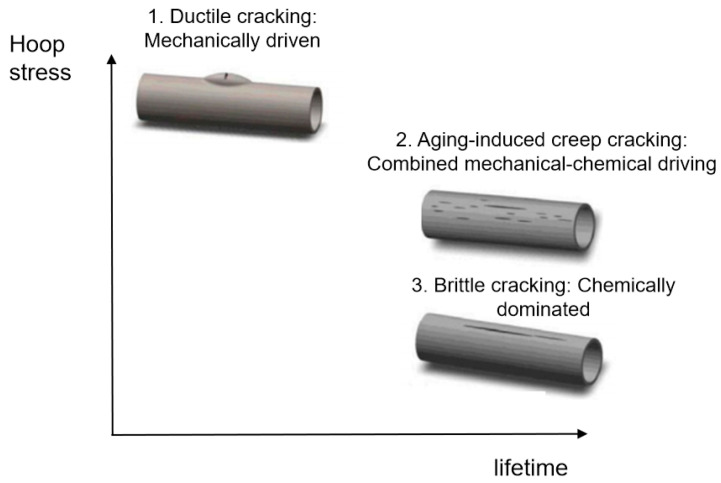
Failure modes of steel wire-reinforced composite pipes [27].

In summary, these results demonstrate that failure initiates with degradation or mechanical damage of the polyethylene layer, which provides pathways for corrosive media to attack the steel wires. This initial failure provides pathways for corrosive media, leading to corrosion of the steel wire skeleton. Although this failure mechanism is consistent, different service conditions and initial material states collectively determine the ultimate failure mode dominated by mechanical, chemical, or coupled mechanochemical factors: ductile cracking, aging-induced brittle cracking, and aging-induced creep cracking (Figure 22), with cracking primarily propagating in the axial direction.

(1) Ductile cracking typically occurs within a few years of service. It is mechanically driven, often triggered by stresses approaching or exceeding the elastic limit of the pipe due to installation damage, external compression, or overpressure. Macroscopically, it manifests as a tear-shaped rupture with significant plastic deformation and wall thinning, as exemplified by the burst failure in Case 1 caused by mechanical gouging and local overpressure.
(2) Aging-induced brittle cracking appears after long-term service (over a decade). Oxidative degradation embrittles the polyethylene molecular structure, leading to slow environmental stress crack propagation under low stress. This chemically dominated failure is exemplified in Case 2, where the pipe failed by brittle perforation after 14 years of service, showing a flat fracture surface, absence of plastic deformation, and extremely low OIT values between 1.4 and 1.9 min.
(3) Aging-induced creep cracking also typically occurs after extended service, resulting from coupled chemical aging and mechanical creep. As in Case 3, the pipe exhibited overall bulging and axial microcracking on the inner surface. Long-term aging degraded the mechanical properties of the polyethylene, and sustained stress induced creep, leading to gradual wall thinning, increased hoop stress, and ultimately, multi-origin crack initiation and propagation.

**Figure 23:**
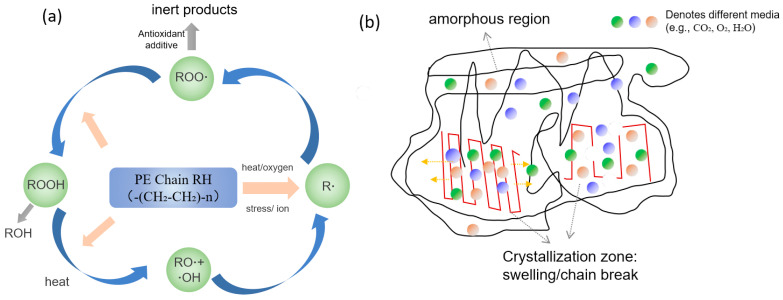
Schematic diagram of polyethylene aging mechanisms: (**a**) aging reaction; (**b**) medium penetration and swelling.

From the perspective of the crack propagation path, the failure of steel wire-reinforced PE composite pipes follows the sequence of degradation of inner and outer PE protective layers → medium penetration and corrosion of steel wire → reduction in pressure-bearing capacity → structural rupture/leakage, with cracks extending through the pipe wall. The inner and outer PE layers act as the primary barrier. Once damaged, the field water medium can infiltrate the pipe body, rapidly corroding and breaking the steel reinforcement. Local structural damage eliminates strength support, enlarges leakage channels, and accelerates through-wall structural failure. The integrity of the PE layer fundamentally determines the service life of the composite pipe. The main causes of PE layer damage include the following:

(1) Poor material quality: low anti-aging performance can result in rapid defect formation under service conditions.
(2) Material aging: according to the literature, PE exhibits good corrosion resistance to H_2_S at 60 °C [18]. However, during long-term service, slow aging is mainly attributed to free-radical chain reactions induced by CO_2_, H_2_O, and O_2_ in high-temperature environments, leading to molecular chain scission. In addition, penetration of organics and Cl^−^ in high-pressure field water causes swelling of the amorphous regions of PE. The cumulative effect of these synergistic damages leads to continuous deterioration of material properties (Figure 23).
(3) External stress or mechanical damage: mechanical analysis shows that the bottom of buried steel wire-reinforced composite pipes bears the highest load [28]. If hard rock layers exist beneath or if uneven settlement occurs, compressive stress on the outer surface may transform into tensile stress on the inner surface, creating local stress concentration. Furthermore, due to the relatively low abrasion resistance of outer PE—especially low-density PE—scratches from contact with sand, gravel, or tools during installation can act as stress concentrators and penetration paths, accelerating chemical aging.
(4) Environmental factors: when service temperatures exceed design limits (typically >65 °C) and pressures exceed design ratings, high temperatures soften PE and relax molecular chains, while high pressures induce severe creep and stress concentration, exacerbating material degradation and failure.

Considering the characteristics, failure modes, and main causes of steel wire-reinforced PE composite pipe degradation, the following protection strategies are proposed:

(1) Materials selection: It is recommended to use high-density polyethylene or heat-resistant (>75 °C) polyethylene as the base resin, increase the loading of antioxidants (e.g., hindered phenols and phosphites), and add wear-resistant additives to the outer-layer resin. In addition, the anti-aging performance of polyethylene should be evaluated more rigorously in conjunction with actual service conditions. For metal reinforcement used in acidic environments, sour-service carbon steel is recommended. Sulfide stress cracking testing per NACE TM0177-2016 [29] and hydrogen-induced cracking testing per NACE TM0284-2016 [30] should be carried out to prevent corrosion cracking of steel wires caused by permeation of sulfur-bearing media, which would accelerate failure.
(2) Installation quality control: Standardize laying and construction technical requirements and strengthen process control during installation to minimize mechanical damage to the pipe wall and avoid contact between hard stones at the trench bottom and the pipe wall that could cause localized stresses. Pipe sections are generally connected using steel couplings; 304 stainless steel is preferred for the coupling material. Sealing gaskets must be installed correctly, with fluororubber (FKM) as the preferred material.
(3) Operational monitoring: Continuously record and monitor pipeline operating parameters, including temperature, pressure, flow rate, and medium composition, and avoid over-temperature and over-pressure operation whenever possible.
(4) Condition-based inspection: Without affecting normal gas field production, periodically (preferably every year) sample in-service pipelines—especially aging pipelines with over ten years of service—during maintenance shutdowns. Conduct material property testing, hydrostatic pressure testing, and joint sealing tests to assess aging and degradation under service conditions and evaluate structural safety.
(5) Failure documentation and analysis: For pipelines that exhibit bulging, rupture, or leakage during service, record the failure details in full and carry out systematic failure analysis to identify root causes.

## 5. Conclusions

(1) Following the failure of the SRTPs, pronounced alterations in its performance metrics were observed. These included deterioration in microscopic morphology, the emergence of carbonyl (C=O) and ether (C–O) functional groups in FTIR spectra, and a marked reduction in oxidation induction time (OIT)—as low as 1.4 min, well below the standard threshold of >20 min. The predominant failure modes comprised mechanically driven ductile fracture, chemically induced brittle cracking attributable to aging, and creep cracking resulting from coupled chemomechanical effects.
(2) The integrity of the polyethylene protective layer is critical to the service life of the SRTP. Once this layer is compromised, gas field water containing H_2_S/CO_2_/Cl^−^ or oxygenated soil media infiltrates the pipe body, causing rapid corrosion of the steel skeleton. This ultimately leads to loss of structural load-bearing capacity and penetrating failure. The main controlling factors include insufficient antioxidant performance of the material, external stress or mechanical damage, and the coupling effect of long-term aging with overpressure or overheating conditions.
(3) It is recommended to systematically develop protective measures in areas such as selection and quality control of anti-aging materials, standardized construction, operational monitoring, and service performance tracking and evaluation to extend the pipeline’s service life and reduce the risk of failure. Future research should focus on establishing a remaining service life prediction model for composite pipes based on key performance parameters such as OIT and hardness.

## Figures and Tables

**Table 1:** Main performance indicators of SRTPs.

Object	Item	Key Performance Indicator	Significance	Test Method
Polyethylene Raw Material	Oxidation Induction Time (210 °C), min	>20	Material aging resistance	GB/T 19466.6 [19]
	Infrared Spectroscopy	Significant changes in new and original peaks	Changes in molecular functional groups; whether decomposition occurs	Fourier Transform Infrared Spectrometer
	Density, kg/m^3^	>930	Whether swelling or decomposition occurs	GB/T 1033.1 [20]
	Carbon Black Dispersion, grade	≤3	UV aging resistance	GB/T 18251 [21]
	Minimum Strength Requirement, MPa	≥8.0	Mechanical properties	GB/T 18252 [22]
carbon steel wire	Appearance	No rust on the surface	Corrosion damage	Visual inspection
	tensile strength, MPa	1720–1970	Mechanical properties	GB/T 228.1 [23]
Pipe Body	Short-Term Hydrostatic Strength	20 °C, 2× nominal pressure, No leakage or rupture within 1 h	Overall sealing performance	GB/T 6111 [24]
	Instant Burst Strength	20 °C, water medium, Instant burst pressure ≥3× nominal pressure	Ultimate internal pressure resistance	GB/T 6111 [24]
	Hardness	Shore Hardness	Resistance to indentation or scratch damage	GB/T 3854 [25]
	Oxidation Induction Time (210 °C), min	>20	Aging resistance	GB/T 19466.6 [19]
Appearance & Structure	Appearance	No obvious scratches, impurities, color unevenness, or other defects	Surface quality	Visual inspection
	Dimensions	Compliance with matching requirements for outer diameter, roundness, and nominal pressure	Overall forming precision	GB/T 8806 [26]

## Data Availability

The original contributions presented in this study are included in the article. Further inquiries can be directed to the corresponding author.
